# Update on the Effects of Antioxidants on Diabetic Retinopathy: In Vitro Experiments, Animal Studies and Clinical Trials

**DOI:** 10.3390/antiox9060561

**Published:** 2020-06-26

**Authors:** Jose Javier Garcia-Medina, Elena Rubio-Velazquez, Elisa Foulquie-Moreno, Ricardo P Casaroli-Marano, Maria Dolores Pinazo-Duran, Vicente Zanon-Moreno, Monica del-Rio-Vellosillo

**Affiliations:** 1 Department of Ophthalmology, General University Hospital Morales Meseguer, 30007 Murcia, Spain; elena.rubio@carm.es (E.R.-V.); elisam.foulquie@carm.es (E.F.-M.); 2 Department of Ophthalmology and Optometry, University of Murcia, 30120 Murcia, Spain; 3 Ophthalmic Research Unit Santiago Grisolia, 46017 Valencia, Spain; 4 Cellular and Molecular Ophthalmolobiology Group, Surgery Department of the University of Valencia, 46010 Valencia, Spain; 5 Red Temática de Investigación Cooperativa en Patología Ocular (OFTARED), Instituto de Salud Carlos III, 28029 Madrid, Spain; rcasaroli@ub.edu; 6 Department of Surgery & Hospital Clinic de Barcelona (IDIBAPS), School of Medicine, University of Barcelona, E-08036 Barcelona, Spain; 7 Institute of Biomedical Research (IIB-Sant Pau—SGR1113), Banc de Sang i Texits (BST), E-08041 Barcelona, Spain; 8 Area of Health Sciences, Valencian International University, 46002 Valencia, Spain; vczanon@universidadviu.com; 9 Department of Anaesthesiology, University Hospital Virgen de la Arrixaca, 30120 Murcia, Spain; monica.delrio@um.es

**Keywords:** diabetic retinopathy, antioxidant, oxidative stress, retina, in vitro, cell, animal, clinical trial, human, patient

## Abstract

Current therapies for diabetic retinopathy (DR) incorporate blood glucose and blood pressure control, vitrectomy, photocoagulation, and intravitreal injections of anti-vascular endothelial growth factors or corticosteroids. Nonetheless, these techniques have not been demonstrated to completely stop the evolution of this disorder. The pathophysiology of DR is not fully known, but there is more and more evidence indicating that oxidative stress is an important mechanism in the progression of DR. In this sense, antioxidants have been suggested as a possible therapy to reduce the complications of DR. In this review we aim to assemble updated information in relation to in vitro experiments, animal studies and clinical trials dealing with the effect of the antioxidants on DR.

## 1. Introduction

Diabetic retinopathy (DR) is one of the most frequent causes of blindness in the adult and elderly population worldwide [1]. Control of blood glucose, blood pressure and lipidemia have been shown to alleviate the appearance and evolution of DR [2]. Nonetheless, some patients might exhibit a progression of DR with appropriate blood glucose and pressure control.

DR is primarily a microvascular disorder related to the loss of pericytes, endothelial cell proliferation, the disruption of tight junctions between endothelial cells, the thickening of the basement membrane, the leakage of fluid and macromolecules from the vessels, and neovascularization [3,4]. All these phenomena imply the damage of non-vascular cells of the retina such as neuronal and glial cells, with deleterious implications for visual function due to macular edema, vitreous hemorrhage or tractional retinal detachment. These events have been associated directly with oxidative stress [3,4].

Otherwise, standard therapies to treat DR (vitrectomy, photocoagulation, intraocular injections of anti-vascular endothelial growth factors (VEGFs) or corticosteroids) are not able to control DR progression in all cases. The difficulty to stop DR is related to the metabolic memory and this phenomenon has also been associated with oxidative stress [3]. These considerations about DR have led one to consider different therapeutic approaches, such as antioxidant supplementation, to rebalance the excess free radical production and/or the defect of antioxidant natural systems. In fact, a number of studies dealing with retinal cells cultured under hyperglycaemic conditions, animal models of DR and clinical trials with diabetic patients have been performed to test the effect of different antioxidants, alone or in combination, on the functional or structural alterations of DR.

## 2. In Vitro Experiments

Evidence observed for in vitro approaches has allowed one to conclude reliable findings on the biological effects of the antioxidant active compounds. These mainly act to alleviate different related events (such as autophagy, inflammatory pathways, apoptosis, angiogenesis and oxidative disbalance) in the pathophysiology of diabetic retinopathy (DR) [4,5,6]. Several studies conclude that antioxidants are able to prevent or even ameliorate DR status, acting against inflammation and oxidative stress. These pathways play a major role in the appearance and clinical evolution of the disease [7,8,9,10].

An environment that mimics DR conditions is hard to emulate in vitro. The presence of multifactorial events with an inflammatory response and oxidative disbalance in the retina (capillary endothelial cells, pericytes, glia and neurosensory retinal cells) lead to a progressive cellular degeneration process with metabolic alterations of the retinal pigment epithelium (RPE) cells, and a dysfunction of the retinal blood barriers (internal and external). The final consequence is the loss of vision [8,11,12]. Hyperglycemia also induces mitochondrial dysfunction with an intracellular and extracellular augmentation of the reactive oxygen species (ROS). In this sense, several intracellular signalling pathways share the inflammatory and oxidative pathways in diabetes, such as polyol and protein kinase C (PKC), p38 MAPK and others [6]. Likewise, it has been observed that epigenetic adjustments are engaged with oxidative stress. The phenomenon, described as metabolic memory, is related to the deleterious effects on tissues caused by hyperglycaemia, even though there are strict glycemic controls. Thus, pathways related to regulatory role of microRNAs, histone modifications, DNA methylation and the sirtuins-histones function can act as epigenetic modifiers and modulators [4,13,14].

The metabolic status of the retina is normally isolated from the rest of the body by the blood–retinal barrier that is made up of an internal barrier by the endothelial capillaries and an external barrier by the RPE cells. In addition, these barriers maintain a state of “immune privilege”, with the ability to modulate the response to different external attacks independently [11,15]. In situations of prolonged hyperglycemia, the metabolic stress involves an insult to the retinal capillary network, its barrier systems, and the highly specialized neural connections of the retina, responsible for vision processes [16,17].

It is hard to emulate hyperglycemia experimentally under in vitro conditions. However, the approaches in which in vitro cell cultures are studied present an excellent efficiency and other various advantages, such as the precision of experimental conditions, and the possibility of mimicking alternative scenarios and concentrations of substances. Despite the additional difficulties of interpreting and extrapolating results in vitro, evidence related to antioxidant properties in settings similar to the DR have played a guiding role in the development of conclusive clinical trials.

The results obtained so far have allowed us to conclude that several of the microvascular changes observed in DR can be prevented or mitigated by different antioxidant medicinal derivatives of plant origin. The properties of resveratrol—a powerful antioxidant, which is anti-inflammatory and neuroprotective—are widely known in in vitro studies, which have subsequently been confirmed in different clinical trials [5]. Other flavonoid polyphenols (e.g., naringin, anthocyanins) and non-flavonoids (e.g., curcumin), are characterised by a group of natural active compounds, of which the antioxidant and anti-inflammatory capacity was verified in human cell models of retinal endothelial cells (HREC) and retinal pigment epithelial cells (ARPE-19), under conditions of sustained high concentrations of glucose, mimicking a diabetic environment [18,19].

Non-provitamin A carotenoids (lutein, zeaxanthin, lycopene, and astaxanthin) were shown to be effective, not only in reducing in vitro inflammation, but also in modulating gene expression, eliminating ROS as a consequence [20]. Furthermore, there is strong evidence for xanthophyl carotenoids, such as lutein and zeaxanthin, which are selectively absorbed by RPE cells, and accumulate preferentially in the macular region, conferring protection on photoreceptors. Recently, it was found that this beneficial effect on the retina can be increased synergistically when lutein is administered in association with Ω-3 long-chain polyunsaturated fatty acids. An antioxidant and anti-inflammatory effect were found by reducing ROS production and inhibiting the expression of inflammatory mediators [21]. In this sense, the fish oil emulsion, rich in Ω-3 polyunsaturated fatty acids, has anti-inflammatory and antioxidant in vitro properties, with the capability to inhibit the production of pro-inflammatory growth factors and cytokines [22]. Table 1 summarizes the main in vitro studies on antioxidants and their results as possible therapeutic agents for DR treatment [22,23,24,25,26,27,28,29,30,31,32,33,34,35,36,37,38,39,40,41,42,43,44,45,46,47,48,49,50].

## 3. Animal Studies

There is an increasing body of evidence about the role of antioxidants in the control of diabetic retinopathy in animal models (Table 2) [51,52,53,54,55,56,57,58,59,60,61,62,63,64,65,66,67,68,69,70,71,72,73,74,75,76,77,78,79,80,81,82,83,84,85,86,87,88,89,90,91,92,93,94,95,96,97,98,99,100,101,102,103,104,105,106,107,108,109].

Vitamins have a determining task in the control of the oxidative cascade involved in the development of diabetic retinopathy [102,103,104,105]. Vitamins C (ascorbic acid) and E [α-tocopherol] have been shown to have the ability of avoiding abnormalities of ocular blood hemodynamics and leukostasis in streptozotocin-induced diabetic rats [102,103]. Moreover, they prevent the advancement of acellular capillaries [102,103,104,105].

Combinations of vitamins have a synergistic effect. In this manner, vitamin E + selenium and taurine diminish retinal conjugated dienes (CD) in the early stage of the diabetic disease. This combination can also reduce lipid hydroperoxides (LP), but with a 500 IU dose of vitamin E [97].

In addition, vitamins C and E, supplemented with other antioxidants (Trolox, *N*-acetyl cysteine, β-carotene and selenium), improve the survival of retinal cells more obviously. This combination prevents a decrease in SOD, glutathione reductase (GR) and catalase, and lowers the activation of retinal protein kinase C and diminishes lipid peroxidation indicators. [102,103].

Trolox is an analog of vitamin E that allows the regeneration of diminished pericytes in retinal vessels in diabetic mice [33].

Age-related eye disease study (AREDS)-based micronutrients (ascorbic acid, vitamin E, β-carotene, zinc, and copper) diminish oxidative and nitrative damage to retinas in diabetic rats and prevent the development of retinal acellular capillaries [52].

Nicanartine (also known as l-carnitine) is responsible for transporting fatty acids into the mitochondria. It also acts as an antioxidant and lipid-lowering compound. Nicarnitine slows down pericyte loss, but has no effect on the formation of capillaries or micro-aneurysms in diabetic rats [86]. Another study showed that l-carnitine can reduce glycemic levels. Administrated daily, it has an important role as an antioxidant factor and avoids protein degradation [77].

High levels of VEGF protein concentration are observed in diabetic rats if compared with control non diabetic rats. Taurine and α-lipoic acid alleviate VEGF levels in diabetic rats [36] and α-lipoic acid maintains a constant number of pericytes and foil the formation of acellular capillaries [54,55,57]. These molecules obtain the inhibition of lipid peroxidation, increase glutathione peroxidase and produce an activation of AMP-activated protein kinase (AMPK) [57]. The normalization of nuclear transcriptional factor and the restoration of antioxidant defences are observed in the retinas of diabetic rats [54,55,56,57]. Moreover, oral supplementation with lipoic acid allows one to re-establish the electroretinogram b-wave amplitude of diabetic animals, to control values [56].

It has been known for a long time that food plays a crucial function in the control of diabetes. However, it is not only necessary to avoid foods with a high glycemic index. Several studies are being carried out to prove the beneficial effect for the control of pathogenesis of structural damage caused by diabetes.

The antioxidant agents present in commonly consumed food have shown beneficial effects. Some examples are blueberry anthocyanins [59], Litchi chinensis [78], Morus alba (white mulberry) [83] Crocin (Saffron) [67] and Sesamin [95].

Hydroxytyrosol is the main polyphenol compound present in olive oil. It has a neuroprotective effect on the retina. In diabetic rats treated with hydroxityroxol, the total retinal thickness and the cellular size were similar to non-diabetic animals. Retinal ganglion cell loss is smaller in rats treated with hydroxytyrosol, compared with non-treated diabetic rats [76].

Curcumin, a natural yellow pigment used commonly to color foods and cosmetics, decreases oxidative stress, diminishes levels of VEGF, NF- KB AND IL-1 β, and seems to have an anti-inflammatory effect on DR [68,69].

Resveratrol is present in the skin of grapes, blueberries, raspberries and blackberries. It is also found in high concentrations in red wine.

A daily dose of Resveratrol suppresses endothelial nitric oxide synthase production [89], the expression of oxidative biomarkers and superoxide dismutase capacity in the retina and blood of diabetic rats [90]. Furthermore, it improves the expression of the PON1 gene that regulates inflammatory response and microvascular complications in diabetes [91] and can restore the transcription of the proteins of the retinoic acid metabolism pathway [92]. Resveratrol coated gold nanoparticles redevelop the equilibrium between the stimulators and inhibitors of antiangiogenesis, increasing the retinal expression of PEDF and decreasing VEGF-1. Retinal expressions of TNFα, IL-1 β, MCP-1, ICAM-1 and IL-6 are also diminished [91,92,93]. Caffeic acid hexyl (CAF6) and Dodecyl (CAF12) amides also have a neuroprotective effect [57,60].

Another antioxidant, Calcium Dobesilate (Ca Dob), inhibits acellular capillaries and pericyte loss, and [61] protects the blood-retinal barrier by preserving tight junctions [62], and induces an important decrease of pro-oxidative markers [61,62,63].

Carotenoids, such as lutein or zeaxanthin, also seem to have protective effects on DR in diabetic rats. Zeaxanthin inhibits oxidative damage and performs anti-inflammatory action [106]. Plus, lutein normalizes the markers of retinal oxidative stress, avoids ganglion cell loss and decreases apoptosis markers (caspase-3) [79]. Furthermore, it attenuates the loss of ganglion cells and prevents impairment in ERGs [79].

Tempol, a superoxide dismutase (SOD) mimetic and pleiotropic intracellular antioxidant, can neutralize the excess of superoxide radical expression in DR. Tempol protects cells of the vessels, especially ameliorating hemodynamics in the retina of diabetic rats [100]. Moreover, tempol avoids the gathering of fibronectin and glial fibrillary acidic protein in a diabetic murine model [100].

*N*-acetylcysteine helps to regulate pro-oxidative markers (ROS, VEGF and ICAM-1) [84]. Apocyn ameliorates and downregulates the TLR4/NF- B pathway activity, prevents microglial activation and stops neuronal autophagy [51]. 

Eriodictyol, a flavonoid obtained from California Yerba Santa, preserves the blood-retinal barrier, decreasing pro-oxidative biomarkers as well [72]. Rutin has shown antiapoptotic activity (decreasing levels of caspase-3) [94]. It has activity at the systemic level, decreasing glucose levels and improving insulin concentration. Plus, other flavonoids with beneficial effects are naningerin [85] and taxifolin, a flavonone found in onion, milk thistle (carduus marianus), douglas fir bark and frech maritime (pinus pinaster) fir bark. Taxifolin is useful to prevent diabetic retinal damage. It can normalize the Il-6, TNF, IL1 and tGSH levels in blood serum [98]. Other flavonoids such as hesperetine [75] have been studied, obtaining promising results.

Green tea, rich in polyphenols, decreases the number of pericyte ghosts and acellular capillaries and forces a lower concentration of VEFG and TNF-α in the retina of animal models [73,74].

Melatonin is a strong antioxidant naturally secreted by the pineal gland. When orally administered, it presents the amelioration of cytokine, nitrotyrosine, and malondialdehyde concentrations, [80] and depletion in concentrations of VEGF, MMP9, and oxidation protein products (AOPP) [81]. Merhazadi et al. also found that oral melatonin administered to diabetic rats decreased retinal cell size and the fluorescein leakage of retinal vessels [82].

## 4. Clinical Studies

There are not as many clinical studies as in vitro or animal investigations. There is an increased interest in antioxidant supplementation for human disorders, because they can be administered orally and they are easily available and affordable. Difficulties in analyzing these studies are that end-points and results are variables and, in some cases, controversial. Some studies focus their objectives on functional results (best-corrected visual acuity—BCVA—; contrast sensitivity; glare sensitivity), others in anatomical results (central macular thickness—CMT—), in analyzing oxidative stress status, or even in a combination of the anterior parameters. Plus, in the majority of these studies, the population studied include type 2 diabetic mellitus (T2DM) patients. Some studies deal with the first stages of the disease and other studies consider typical complications of late stages. Furthermore, as previously noted, tangled and not well-known mechanisms in the pathogenesis of diabetic retinopathy make early and new combined therapies of antioxidants desirable. The most relevant studies are listed in Table 3.

Table 3 shows three studies with ALA. Single administrations in 467 T2DM patients over the course of two years found no results preventing diabetic macular edema (DME) or improving BCVA. [107,108]. ALA combined with genistein and vitamins C, E and B in 32 pre-retinophatic patients over the course of 30 days showed some increases in plasma antioxidant levels and eletroretinogram (ERG) oscillatory potential values [119].

Calcium dobesilate (Ca Dob) is a vasculoprotector agent that has been widely studied in the context of DR. [138]. However, the results of clinical trials in DR are controversial. Farsa [139] described some Ca Dob mechanism of actions, such as antioxidant and anti-ROS activity, defensive activity at the endothelial level, antiapoptoic properties and angiogenesis inhibition. Other mechanisms of Ca Dob improving microcirculation and reducing micro-vascular injury have been described recently [140]. There are nine studies listed with Ca Dob supplementation (Table 3). The study with the highest number of patients recruited (635) and the longest duration showed no effects on the reduction of the development of clinically significant diabetic macular edema (CSDME) in T2DM [116]. Likewise, another study with 40 non-proliferative DR (NPDR) patients with macular edema failed to find beneficial effects by adding Ca Dob to laser in the treatment of macular edema [117]. However, other studies have positive results analyzing microaneurisms and retinal hemorrhages [115], exudates [113], and blood viscosity [111,112].

There is an increasing interest in combined antioxidant therapy (CAT), as shown by the latest investigations. A long-lasting study (60 months) by Garcia-Medina et al. [118] evaluated antioxidant supplementation (lutein, VC, alpha-tocopherol, niacin, beta-carotene, Zn and Se) in 105 T2DM with NPDR. This long follow-up allowed one to demonstrate a retardation of the DR progression and the maintenance of antioxidant plasma status in the treated group. However, no effect on visual acuity was reported. In another work, the supplementation of lutein and zeaxanthin for three months in type 1 diabetic mellitus (T1DM) and T2DM showed an improvement of visual acuity, contrast sensivity and foveal thickness [127]. Likewise, Chous et al. reported an improvement in BCVA, but no changes in retinal thickness in his study. They administered a DiVFuSS formula that consists of vitamins C, D3 and E (d-α tocopherol), zinc oxide, eicosapentaenoic acid, docosahexaenoic acid, α-lipoic acid (racemic mixture), coenzyme Q10, mixed tocotrienols/tocopherols, zeaxanthin, lutein, benfotiamine, N-acetyl cysteine, grape seed extract, resveratrol, turmeric root extract, green tea leaf, and Pycnogenol [122].

Other studies focus their results on the interpretation of oxidative/nitrosative stress and antioxidant defenses, such as Rodriguez-Carrizalez in a trial with 60 T2DM patients over the course of months [123]. Furthermore, Roig Revert et al. studied 208 T2DM for 18 months and found a reduction of MDA and a significant increment of total antioxidant status (TAS), a reduction of MDA and a significant increase of TAS in the treated retinopathy group [121].

The association of CAT with the latest therapies such as anti-VEFG therapy is very interesting. Lafuente et al. [124] studied 55 T2DM for three years. They measured the effect of adding a combined antioxidant treatment to ranibizumab in the treatment of diabetic macular edema (DME). They reported lower macular thickness in the supplement group when compared to the control group.

The antioxidants, anti-inflammatory and neuroprotective effects of crocin have also been studied by Sepahi et al., in 60 DM pattens with refractive DME showing promising results [125].

Ginkgo biloba supplementation has been tested by Huang in 25 T2DM patients for three months. They found a decrease in MDA and fibrinogen levels and an improvement in other blood parameters and retinal blood flow rate [126].

Pycnogenol^®^, a French maritime pine bark extract rich on flavonoids, has been dosed alone or combined since the 1980s, in several studies. Two studies administering this antioxidant alone (Table 3) are referenced. One is a study of 32 diabetic patients for six months, five of which displayed a reduction in exudates in both eyes [113]. The other study reported the functional and anatomic results of 46 T2DM for three months [131] Domanico et al. have studied patients with NPDR. Patients were divided into two groups: receiving supplementation or placebo. The treatment was made up of an antioxidant combination (pycnogenol, vitamin E and coenzyme Q10). Treated group showed that central macular thickness was significantly reduced during the study period (six months) [120].

In 2020, Ali et al. published an exploratory, two-month investigation, to evaluate the efficacy and safety of resveratrol in T1DM patients. A total of 13 patients were studied. Between the baseline and the endpoint resveratrol, treatment was associated with a significant decrease in the level of MDA and a significant increase in the level of total antioxidant capacity. There was no change in the serum levels of TNF-alpha and IL-1beta [132].

Tabatabaei-Malazy et al. reviewed 10 observational studies that reported lower vitamin C levels in subjects with DR compared to those without DR [141]. Park et al. studied the association of vitreous vitamin C depletion with diabetic macular ischemia in proliferative diabetic retinopathy (PDR). This is a study with 40 patients, 20 with PDR and 20 with idiopathic epiretinal membrane (control group), that underwent a pars plane vitrectomy. They found that a vitreous level of vitamin C in PDR patients showed a decrease, that was correlated with the degree of macular ischemia. Levels of vitamin C in the serum, aqueous humor and vitreous humor were lower in patients with PDR than in the control group. However, there was no correlation among the serum, aqueous humor and vitreous humor levels of vitamin C in the PDR group. These findings suggest that ocular factors could be relevant in the pathogenesis of PDR and open new strategies and routes of administration of the treatment [133].

Two clinical trials administering vitamin E (α-tocopherol) are included (Table 3). Bursell et al. studied high-dose vitamin E supplementation in a T1DM group and in a control group. The authors reported that diabetic patients showed a decreased retinal blood flow that improved after treatment, similarly to control cases [134]. Chatziralli et al. administered 300 mg of vitamin D to 282 insulin dependent T2DM with DR. They found a significant diminution of MDA in all DR stages [135].

Vitamin E supplementation was compared to ALA or selenium supplementation or placebo, in a study performed by Kähler in 80 diabetics during three months. They found that oxidative status was reduced in all treated groups compared to the placebo group [108].

Finally, a recent study by Kheirour analyzed serum VEFG levels with zinc supplementation. The authors did not observe any changes after three months of treatment [137].

## 5. Conclusions

The studies examined in this survey suggest that a number of antioxidants may prevent or alleviate the deleterious effects of hyperglycaemic environment in cultured tissues, or the complications of DR in animal models of diabetes. The results in human studies are more controversial, but some of them are quite promising, particularly when combinations of antioxidants are considered. It is necessary to keep in mind that studies in humans cannot be compared to those performed in cultured tissues with a perfect control of the environment or performed in laboratory animals in which diets and the rest of the living habits are supervised. In addition, metabolism, senescence rate or administered doses in animal models are not identical to those in human individuals [142].

The heterogenicity of the studies included in this review, in terms of antioxidant types, methods, doses and results, is remarkable. It is difficult to bring together all this information, but we can infer that there is a tendency to use antioxidants in combination. However, we have to keep in mind that pro-oxidative conditions and their consequences are still not well-known in a chronic disorder such as DR. Plus, a combination of antioxidant may interact not only with different metabolic pathways, but also among themselves. In this context, it is hard to define the exact effect of each antioxidant that may even change when administered in combination.

Another important concern is the safety of administering antioxidants for years, when the clinical studies performed so far are not longer than five years.

Despite the fact that encouraging outcomes have already been obtained in this field, future studies must be conducted considering the efficacy and safety of different doses of antioxidants, in order to get better results to prevent ocular damages in this blinding disease.

## Figures and Tables

**Table 1:** In vitro studies on diabetic retinopathy (DR) and hyperglycemic conditions (↑ = increase of; ↓ = decrease of).

Antioxidant(s) Studied.	Culture Cell Line Type	Main Outcomes	Reference
Alpha-linolenic acid (ALA), zinc and linoleic acid	Choroid-retina endothelial cells (monkey)	Modulation of endothelial proliferation. ALA ↓ reactive oxygen species (ROS) production and vascular endothelial growth factors (VEGF) secretion, and ↑ superoxide dismutase (SOD) activity.	Shen et al. 2012 [43]
AMG-487	Human endothelial cells	↓ oxidative and endoplasmic reticulum stress	Wang et al. 2019 [48]
Ascorbic acid, α-tocopherol and α-lipoic acid	Bovine endothelial cells of the retina	↓ superoxide anion production	Wu et al. 2012 [44]
Astragaloside-IV	Murine endothelial cells of the retina	↓ mitochondrial ROS. ↓ superoxide and hydrogen peroxide and	Qiau et al. 2017 [30]
β-carotene, lutein and lycopene	Human retinal pigment epithelium (RPE)	↓ cell loss	Gong et al. 2017 [23]
BM-MSC (Bone-marrow mesenchymal stem cells)	Murine ganglion cells of the retina	↑ defensive effect after alterations caused by H2O2, ↓ cytokines, and ↑ neurotrophin secretion	Cui et al. 2017 [25]
Calcium dobesilate	Human cultured veins	↑ total antioxidant status (TAS) and ↓ malondialdehyde	Alda et al. 2011 [42]
dh404 (Nrf2 activator)	Murine Müller cells	↑ NADH/NADPH, Nrf2, quinine oxidoreductase-1 and hemeoxygenase-1	Deliyanti et al. 2018 [32]
EPA and DHA	Human RPE	↓ ROS	Dutot et al. 2011 [40]
Epigallocatechin-3-gallate (EGCG)	Human endothelial cells of the retina	↓ cytokines and apoptosis and	Zhang et al. 2016 [31]
Fidarestat (aldose reductase inhibitor)	Bovine endothelial cells of the retina	↑ antioxidant defenses	Obrosova et al. 2003 [36]
Fish oil emulsion (FOE)	U937 cell line (monocytes/macrophages)	↑ antioxidant proprieties with ↓ pro-inflammatory cytokines, ↓ cellular damage	Laubertová et al. 2017 [22]
Galangin	Human endothelial and RPE cells of the retina	↑ activation of Nrf2, reverse ↓ expression of claudin-1 and occludin, and ↓ ROS formation	Zhang et al. 2019 [50]
He-Ying-Qing-Re Formula (HF)	Murine retinal ganglion cell culture	↓ endoplasmic reticulum stress; ↓ H2O2-induced apoptosis; ↓ mitochondria-related proapoptotic factors	Zhang et al. 2018 [27]
KIOM-79	Murine pericyte cell culture	↓ apoptosis by ↓ ROS production	Kim et al. 2010 [28]
Lignans extract (Eucommia ulmoides)	Choroid-retina endothelial cells (monkey)	↓ oxidant effects by regulating via Nrf2 pathway	Liu et al. 2016 [29]
MnTBAP	Bovine endothelial cells of the retina	↓ mitochondrial DNA insult	Madsen-Bouterse et al. 2010 [38]
*N*-acetylcysteine (+ SS31, mitochondrial antioxidant)	Human RPE	↓ mitochondrial dysfunction, oxidative stress and mitophagic flux to lysosomes induced by Auranofin.	Yumnamcha et al. 2019 [47]
Naringin	Murine Müller cells	↓ inflammatory and pro-oxidant effects	Liu et al. 2017 [26]
PEDF	Bovine pericytes of the retina	↑ glutathione peroxidase; apoptosis; Inhibition of caspase-3	Amano et al. 2005 [37]
Selenium	Human RPE	↓ glutathione peroxidase	González De Vega et al. 2018 [24]
SNJ-1945	Murine retinal ganglion cell culture	↓ apoptosis induced by high glucose environment	Shanab et al. 2012 [45]
SS31	Human endothelial cells of the retina	↓ ROS and caspase-3	Li et al. 2011 [41]
Sulphoraphane	Rat Müller cell line	↓ TNF-α and IL-6 levels; ↑ GSH, SOD, and catalase activities.	Li et al. 2019 [49]
Supplement combined (Vitamin C, Trolox, *α*-tocopherol acetate, N-acetyl cysteine, β-carotene, selenium)	Bovine endothelial cell and pericyte culture	↓ caspase-3	Kowluru et al. 2002 [34]
Supplement (ascorbic acid, Trolox, *α*-tocopherol acetate, N-acetyl cysteine, β-carotene, selenium)	Bovine endothelial cell and pericyte culture	↓ NF-kB and nitric oxides and nitrotyrosine formation	Kowluru et al. 2003 [35]
Taurine	Rat Müller cell line	↓ TBARS, ROS; ↑ GSH-px, catalase and SOD activities in relation to dose	Zeng et al. 2010 [39]
Trolox	Cultured rat retina	↓ TBARS	Ansari et al. 1998 [33]
Vitamin D	Human RPE	↓ ROS and caspase-3/7 activities	Tohari et al. 2020 [46]

**Table 2:** Results of antioxidant supplementation in animal models.

Antioxidant Studied	Outcomes in Treated Animals	Reference
Apocynin ameliorates (medicinal herb Picrorhiza kurroa)	Regulate the inflammation through inhibition od TLR4/NF-kB pathway.	Wang et al. [51] 2019
AREDS-based micronutrients	Avoidance of oxidative and nitrative stress. Prevention of formation of ghost capillaries.	Kowluru et al. [52] 2008
α-lipoic acid or taurine	Improves levels of VEFG and reduces ROS biomarkers.	Obrosova et al. [36] 2001
α-lipoic acid or D-α-tocopherol	Frustrates the increase in leukostasis.	Abiko et al. [53] 2003
α-lipoic acid	Control of retinal lipid peroxidation. Stunting of capillary apoptosis and acellular capillaries.	Kowluru et al. [54] 2004
α-lipoic acid	Control of nuclear transcriptional factor and angiopoietin-2. Reduction of VEGF and ROS species. Avoid pericyte ghost.	Lin et al. [55] 2006
α-lipoic acid	Reestablishment of ERG b-wave amplitude. Avoidance of GSH depletion. Normalization of MDA.	Johnsen-Soriano et al. [56] 2008
α-lipoic acid	Inhibits cells death. Activation od AMP- activated protein kinase (AMPK). Inhibition of O- linked–β-N acetylglucosamine transferase (GOT).	Kim et al. [57] 2018
Aster tataricus	Preservation of vascular permeability. Attenuation of TNFa, IL10 and NF-kB.	Du et al. [58] 2017
Blueberry anthocyanins	Downregulation NRF2 pathway. Reestablishment of VEGF and IL-1β levels.	Song et al. [59] 2016
Caffeic acid hexyl (CAF6) and dodecyl (CAF12) amide derivatives	Increase superoxide dismutase (SOD) activity and iso prostaglandin F2 alpha. Decrease retinal oedema and improve neuronal survival signal.	Fathalipour et al. [60] 2019
Calcium dobesilate	Improvement of vascular tortuosity. Stunting of capillary apoptosis and acellular capillaries.	Padilla et al. [61] 2005
Calcium dobesilate	Avoids blood-retinal barrier breakdown and leukocyte adhesion to vessel wall.	Leal et al. [62] 2010
Calcium dobesilate	Inhibition NF-kB pathway. Reduction of TNF- α IL-6, and MPC-1.	Bogdanov et al. [63] 2017
Calcium dobesilate	Increase of GFAP, attenuation of cytokine expression and increase in oxidised nitrotyrosine and carbonyls.	Voabil et al. [64] 2018
Cannabidiol (CBD)	Diminution of TNF-α, VEGF, ICAM. Maintenance of vascular permeability.	El-Remessy et al. [65] 2006
Carnosine	Vasoprotective effect. Induction of protective Het shock proteins in activated glial cells and normalization of hyperglycemia-induced Ang-2.	Pfister et al. [66] 2011
Crocin (saffron)	Microglial activation. Neuroprotective.	Yang et al. [67] 2017
Curcumin	Improvement of oxidative stress biomarkers.	Kowluru et al. [68] 2007
Curcumine	Restoration of expression and function of DNAmethyltransfere (DNMT).	Maugeri et al. [69] 2018
DHA or lutein	Restoration of ERG b-wave amplitude. Inhibition of lipid peroxidation and apoptosis markers. Improvement of retinal thickness.	Arnal et al. [70] 2009
Ebselen or lutein	Reduction of ROS species.	Miranda et al. [71] 2004
Eriodictyol	Mitigation of retinal inflammation and plasma lipid peroxidation, Preservation of blood-retinal barrier.	Bucolo et al. [72] 2012
Fidarestat	Inhibition aldose reductase pathway.	Obrosova et al. [36] 2003
Green tea/Vitamin C-E	Diminution of ghost pericytes and acellular capillaries. Lower superoxide capacity.	Mustata et al. [73] 2005
Green Tea	Lowering expression of proinflammatory mollecules (VEGF and TNF-α).	Kumar et al. [74] 2012
Hesperetin	Reduction of levels of cytokines. Inhibitory effect on caspase-3, GFAP and AQP4 expression.	Kumar et al. [75] 2013
Hydroxytyrosol [olive oil]	Neuroprotective effect. Slowing down on ganglion retina cell counts. Decrease of retinal thickness and cellular size.	Gonzalez-Correa et al. [76] 2018
l carnitine	Improvement of glucose levels. Inhibitory effect on protein degradation.	Samir et al. [77] 2018
Lichi chinensis	Downregulation of proteins carbonyl subproducts and aldose reductase.	Kilari et al. [78] 2016
Lutein	Avoidance of ganglion cell loss. Reduction of apoptosis markers like caspase-3.	Sasaki et al. [79] 2010
Melatonin	Reduction od retinal nitrotyrosine and malondialdehyde levels, The vasomodulator cytokines are decreased.	Ozdemir et al. [80] 2014
Melatonin	Depletion in concentrations of VEGF MMP9, and oxidation protein products (AOPP).	Djordjevic et al. [81] 2018
Melatonin	Decreased fluorescein retinal leakage, ROS and malondialdehyde levels.	Mehrzadi et al. [82] 2018
Morus Alba	Reduces glucose levels and VEGF levels. Inhibition polyol pathway.	Mahmoud et al. [83] 2017
*N*-acetylcysteine	Restoration VEGF and ICAM-1. Diminution of free radicals.	Zhu et al. [84] 2012
Naringenin	Controls glucose levels, increases insulin. and retinal glutathione.	Al-Dosari et al. [85] 2017
Nicanartine	Prevention of endothelial proliferation and pericyte loss.	Hammes et al. [86] 1997
Obtisofplin	Improvement of capillary cell apoptosis and the number of acellular capillaries in the retina.	Hou et al. [87] 2014
PEDF	Restoration of amplitudes of a- and b-wave of ERG; reduced retinal VEGF; reduction of retinal 8-hydroxydeoxyguanosine, a marker of oxidative stress. inhibition of retinal vascular hyperpermeability.	Yoshida et al. [88] 2009
Resveratrol	Inhibition of nitric oxide synthase in endothelial cells.	Yar et al. [89] 2012
Resveratrol	Strengthening of oxidative markers (lipid peroxidation index and oxidized to reduced glutathione ratio) and superoxide dismutase activity in blood and retina.	Soufi et al. [90] 2012
Resveratrol	Recovers insulin level. Improve paraoxonase 1 (PON1) gen activity, reducing vascular permeability and of VEGF, TNF- α, MPC-1, IL-6 IL-1β, INFγ levels.	Chen et al. [91] 2018
Trans resveratrol	Reduces vascular lesion, NF-kB and TNF- α. Stimulates the expression of Ndf2 and SIrT1 genes.	Al Hussaini et al. [92] 2018
Resveratrol coated gold nanoparticles	Decrease expression of VEGF, TNF- α, MPC-1, ICAM 1, IL-6 and IL-1β. Restore balance between inhibitors and stimulators of angiogenesis.	Dong et al. [93] 2019
Rutin	Decrease of glutatione, brain-derived neurotrophic (BDNF), nerve growth factor and caspase.	Ola et al. [94] 2015
Sesamin	Improves blood glucose levels and body weight. Reduces ROS levels and inflammatory biomarkers.	Ahmad et al. [95] 2016
Shikimic Acid (SA) (Artemisia absinthium)	Reduces glucose and glycated hemoglobine levels. Decreases IL-1β and TNF- α.	Al Malki et al. [96] 2019
Taurine/vitamin E+selenium	Diminished conjugated dienes in retina at the early stage of diabetic retinopathy. Reduced lipid hydroperoxides.	Di Leo et al. [97] 2003
Taxifolin	Reduction of total glutathione level. Decrease MDA, IL-1β and TNF- α blood levels.	Ahiskali et al. [98] 2019
Tempol	Improvements in retinal microvascular hemodynamics and blood flow rates.	Yadav et al. [99] 2011
Tempol	Lowers oxidative stress, fibronectin and glial fibrillary acidic protein.	Rosales et al. 2011 [100]
Trigonella foenum	Decreases of inflammatory and angiogenic markers (TNF-α, VEGF, IL1-β).	Gupta et al. [101] 2014
Trolox	Avoids pericyte loss.	Ansari et al. 1998 [33]
Vitamins C and E	Less acellular capillaries and pericyte ghosts.	Kowluru et al. 2001 [102]
Vitamins C and E	Prevent formation of acellular capillaries. Reduce pericyte ghost cells.	Yatoh et al. [103] 2006
Vitamin C	Suppression of leukocyte adhesion. Increase iris blow flow perfusion.	Jariyapongskul et al. [104] 2007
Vitamin	Prevention of blood retinal barrier breakdown. Diminution of VEGF, ICAM1, TNF- α, SOD, IL-1, IL-6 and aldose reductase.	Kunisaki et al. [105] 1998
Zeaxanthin	Reduction of Oxidative damage.	Kowluru et al. [106] 2008

**Table 3:** Results of human studies. CAT = combined antioxidant therapy.

Oral Antioxidant Studied	Recruited Patients [n]	Outcomes in Treated Patients	Follow-Up	Reference
Alpha lipoic acid	467	No results preventing clinically significant macular edema in T2DM. No improvement in best-corrected visual acuity.	2 years	Haritoglou et al., 2011 [107]
Antioxidant combination: Alpha lipoic acid or Selenium or Vit. E	80	All treated groups showed decreased blood TBARS levels and urinary albumin excretion rates.	3 months	Khahler et al., 1993 [108]
Calcium Dobesilate	18	No influence on the capillary resistance of diabetic retinopathy	8 months	Larsen et al., 1977 [109]
Calcium Dobesilate	42	No results in non-proliferative diabetic retinopathy	42 [6 months] 36 [1 year]	Stamper et al., 1978 [110]
Calcium Dobesilate	50	Treated DM + glaucoma patients showed decrease of capillary fragility, blood viscosity and microvascular hyperpermeability.	3 months	Vojnikovic et al., 1984 [111]
Calcium Dobesilate	37	Decrease of whole blood viscosity and capillary fragility.	3 months	Benarroch et al., 1985 [112]
Calcium Dobesilate [Dexium^®^] or Pycnogenol^®^	32	Both drugs improved exudates, Dexium^®^ only in 1 case. Both drugs, particularly Pycnogenol^®^, showed improvements on parameter of automated visual field.	6 months	Leydhecker et al., 1986 [113]
Calcium Dobesilate	79	Non-insulin dependent diabetics showed reducing of whole blood and plasma viscosity and retinal hemorrhages.	6 months	Vojnikovic, 1991 [114]
Calcium Dobesilate	137	In T2DM, better improvement than placebo on microaneurysms, DR level and retinal hemorrhages.	2 years	Ribeiro et al., 2006 [115]
Calcium Dobesilate	635	No effects reducing development of clinically significant macular edema in T2DM	5 years	Haritoglou et al., 2009 [116]
Calcium Dobesilate	40	NPDR with macular edema received laser +placebo or laser + Ca Dob. This study showed no statistically significant difference in macular thickness between Doxium and placebo.	6 months	Feghhi et al., 2014 [117]
CAT formulation [Vitalux Forte^®^]	105	No effect on visual acuity. T2DM treated group show retardation of progression and maintainance of antioxidant plasma status level and decreased plasmatic MDA.	5 years	Garcia-Medina et al., 2011 [118]
CAT: Alpha lipoic acid +genistein+Vitamins C, E and B.	32	Pre-retinopathic diabetics treated group showed increases ERG oscillatory potencial values and plasma antioxidant levels.	30 days	Nebbioso et al., 2012 [119]
CAT: Coenzyme Q10, Pycnogenol^®^, Vitamin E.	68	Treated T2DM: significantly reduced free oxygen radical test levels and CMT.	6 months	Domanico et al., 2015 [120]
CAT: DHA, glutathione, hydroxytyrosol, vitamins E, C, B1, B2, B3, B6, B9, B12, lutein, zeaxanthin, Se, Mn, Zn, Cu.	208	Reduced MDA, significant increased TAS in treated T2DM with diabetic retinopathy group.	18 months	Roig Revert et al., 2015 [121]
CAT [DiVFuSS formula]	67	Better visual function. No effect on macular thickness.	6 months	Chous et al., 2016 [122]
CAT or Coenzyme Q10	60	Lower ROS expression (LPO, nitrites/nitrates) and augment antioxidant defences.	6 months	Rodriguez-Carrizalez. 2016 [123]
CAT: DHA, EPA, vitamins B, C, E and Zeaxantin	55	RD patients with macular edema treated with ranibizumab. Thinner macular in the supplemented group.	3 years	Lafuente et al., 2018 [124]
Crocin	60	Patients with refractory DME divided in 3 groups: 5 mg crocin, 15 mg crocin and placebo. 15 mg crocin group showed significant reduction of HbA1c and CMT and increase of BCVA compared with placebo group	9 months	Sepahi S et al., 2018 [125]
Ginkgo biloba extract	25	Decrease of MDA and fibrinogen levels. Improved blood parameters (viscosity, viscoelasticity, red blood cell deformability, and retinal blood flow).	3 months	Huang et al., 2004 [126]
Lutein + zeaxanthin	90	Treated group showed improvement of visual acuity, contrast sensitivity and reduction of foveal thickness.	3 months	Hu BJ et al., 2011 [127]
Lutein	31	Treated NPDR showed improvement at low spatial frequency in contrast sensitivity.	9 months	Zhang PC et al., 2017, [128]
Lutein + zeaxanthin	72	T2DM NPDR patients 36 received L and 36 received L+ Z. No significant differences in visual acuity, contrast sensitivity and glare sensitivity.	4 months	Yongo-bo et al., 2019, [129]
Pycnogenol^®^	30	Treated group showed not worsening of retinal retinal function and visual acuity.	2 months	Spadea et al., 2001 [130]
Pycnogenol^®^	46	Improvement of visual acuity, flow at the central retinal artery. Reduction in retinal thickness. In T2DM.	3 months	Steigerwalt et al. 2009 [131]
Resveratrol	13	T1DM showed significant decreased levels of MDA and increased total antioxidant capacity between baseline and endpoint. There was no change in the serum levels of TNF- alpha and IL-1beta.	2 months	Ali Movahed et al. 2020, [132]
Vitamin C	40	Vitreous levels of vitamin C in PDR patients showed a tenfold decrease, which was associated with degree of macular ischemia.		Park SM et al., 2019, [133]
Vitamin E	45	Diabetic patients showed decreased retinal blood flow that improved after treatment similarly to non-diabetic cases.	8 months	Bursell et al., 1999 [134]
Vitamin E	282	Decrease of MDA.	3 months	Chatziralli et al., 2017 [135]
Zinc	18	Patients with retinopathy showed increasement of plasmatic GSH-px activity. All patients showed decrease of TBAR.	3 months	Faure et al., 1995 [136]
Zinc	45	DM patients had a negative correlation between serum VEFG levels and Zinc. Treated group showed no changes in VEGF levels.	3 months	Kheirouri et al., 2019, [137]
